# Bimodal In Situ Analyzer for Circular RNA in Extracellular Vesicles Combined with Machine Learning for Accurate Gastric Cancer Detection

**DOI:** 10.1002/advs.202409202

**Published:** 2025-01-17

**Authors:** Yuhang Guo, Shihua Luo, Sinian Liu, Chao Yang, Weifeng Lv, Yuxin Liang, Tingting Ji, Wenbin Li, Chunchen Liu, Xin Li, Lei Zheng, Ye Zhang

**Affiliations:** ^1^ Department of Laboratory Medicine Guangdong Provincial Key Laboratory of Precision Medical Diagnostics， Guangdong Engineering and Technology Research Center for Rapid Diagnostic Biosensors， Guangdong Provincial Key Laboratory of Single Cell Technology and Application School of Laboratory Medicine and Biotechnology Nanfang Hospital Southern Medical University Guangzhou Guangdong 510515 P. R. China; ^2^ Center for Clinical Laboratory Diagnosis and Research Key Laboratory of Research on Clinical Molecular Diagnosis for High Incidence Diseases in Western Guangxi of Guangxi Higher Education Institutions Affiliated Hospital of Youjiang Medical University for Nationalities Baise Guangxi 533000 P. R. China; ^3^ Department of Laboratory Medicine Foshan Hospital of Traditional Chinese Medicine, The Eighth Clinical Medical College of Guangzhou University of Chinese Medicine Foshan Guangdong 528000 P. R. China

**Keywords:** bimodal detection, circular RNA, DNA nanotechnology, early cancer diagnostics, machine learning

## Abstract

Circular RNAs in extracellular vesicles (EV‐circRNAs) are gaining recognition as potential biomarkers for the diagnosis of gastric cancer (GC). Most current research is focused on identifying new biomarkers and their functional significance in disease regulation. However, the practical application of EV‐circRNAs in the early diagnosis of GC is yet to be thoroughly explored due to the low accuracy of EV‐circRNAs analysis. In this study, a hybridization chain reaction system based on rectangular DNA framework guidance and constructing a bimodal EV‐circRNA in situ analyzer (BEISA) is developed. The analyzer can provide dual signal outputs in the fluorescence and electrochemical modes, enabling a self‐correcting detection mechanism that significantly improves the accuracy of the assay. It has a broad detection range and an extremely low limit of detection. In a clinical cohort study, the BEISA used four circRNAs as biomarkers, combining them with machine learning for multiparametric analysis, which effectively differentiated between healthy donors and patients with early‐stage GC. It is believed that the BEISA, in conjunction with machine learning technology, provides an efficient, sensitive, and reliable tool for EV‐circRNA analysis, aiding in the early diagnosis of GC.

## Graphical abstract

They developed a bimodal EV‐circRNA in situ analyzer (BEISA) using a DNA rectangular frame‐guided hybridization chain reaction (RF‐HCR) system. By combining ML algorithms and bimodal EV‐circRNA in situ analysis, this platform provides a promising tool for the analysis of gastric cancer‐derived EV‐circRNA, making diagnosis and staging of gastric cancer possible.

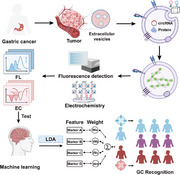

## 1 Introduction

Gastric cancer (GC) is cancer characterized by high morbidity and mortality rates. It is the fourth most common malignant tumor and the fourth leading cause of cancer‐related deaths in men worldwide, posing a great challenge to public health.^[^ ^1^, ^2^ ^]^ Patients with early‐stage GC have a five‐year survival rate of 30–70% after surgery, whereas those with progressive GC have significantly lower rates of only 3–20%.^[^ ^3^, ^4^ ^]^ Therefore, the early diagnosis of GC is important.^[^ ^5^, ^6^ ^]^ Existing methods for detecting GC are mainly based on imaging techniques such as PET/CT and gastroscopy, with tissue biopsy serving as the gold standard.^[^ ^7^, ^8^ ^]^ These methods are invasive, tedious, time‐consuming, and require specialized procedures, often resulting in poor sensitivity and specificity. Therefore, there is an urgent need to establish a liquid biopsy method to facilitate the early diagnosis of GC, which is essential for improving long‐term clinical prognosis.^[^ ^9^, ^10^, ^11^, ^12^ ^]^

Extracellular vesicles (EVs) are membranous structures encapsulated by a lipid bilayer measuring 30–200 nm in diameter and are produced by nearly all cell types.^[^ ^13^, ^14^ ^]^ These vesicles contain a range of biomolecules, including lipids, proteins, and nucleic acids. They are crucial for intercellular communication and are involved in various physiological and pathological processes.^[^ ^15^, ^16^, ^17^ ^]^ EVs are particularly abundant in noncoding ribonucleic acids (RNAs), such as circular (circRNAs), long noncoding, and micro‐RNAs, which play a significant role in regulating the signaling pathways of target cells and are closely linked to the development of numerous diseases.^[^ ^18^, ^19^ ^]^ Among these RNA types, EV‐circRNAs are differentially expressed in patients with early‐stage GC compared with healthy individuals (HDs). Furthermore, because of the protective characteristics of the phospholipid bilayer, it exhibits greater stability in the bloodstream than circulating circRNAs, thus facilitating its detection in the blood and other bodily fluids. Consequently, EV‐circRNAs are viewed as promising cancer biomarkers, offering new insights into the diagnosis of GC.^[^ ^20^, ^21^, ^22^ ^]^

Current research on EV‐circRNAs focuses on identifying new biomarkers and their functional significance in disease regulation. For example, Zhang et al. isolated and purified circRNAs from the plasma exosomes of 80 GC patients and HDs, respectively, and determined that the expression level of circNRIP1 was significantly elevated in the plasma of GC patients.^[^ ^23^ ^]^ Xie et al. demonstrated that EV‐circSHKBP1 in GC patients adsorbed miR‐582‐3p through sponges and interacted with HSP90, thus playing a pro‐cancer role.^[^ ^24^ ^]^ However, the detection and analysis of EV‐circRNAs still face many challenges, including the wide variety of EV‐circRNAs with complex mechanisms of action,^[^ ^25^ ^]^ significant heterogeneity in expression patterns across patients,^[^ ^26^ ^]^ the need for assays that detect low‐abundance markers in early‐stage disease, and the necessity of maintaining stable and reliable sample processing. These factors make it difficult to interpret the complex and vast data related to EV‐circRNAs. To overcome this, there is a need for highly stable and reliable detection technologies that can effectively synthesize and analyze data from multiple sources.

In situ analysis of EV‐circRNAs enables the direct detection of signals from targeted circRNAs in EVs by loading specific probes into individual EVs. In contrast to the gold standard for RNA detection (RT‐qPCR), in situ analysis avoids the need for EV lysis or RNA extraction, preventing sample loss during these processes and providing a high‐quality database for subsequent data analysis.^[^ ^27^, ^28^, ^29^, ^30^, ^31^, ^32^ ^]^ However, current in situ EV‐RNA detection methods, although avoiding EVs lysis and RNA extraction, usually rely on a single signal output, which may lead to insufficient detection sensitivity or stability,^[^ ^33^, ^34^ ^]^ especially in the detection of low‐abundance EV‐RNAs, and may be difficult to provide comprehensive EV‐circRNA characterization. Machine learning (ML) algorithms demonstrated an excellent ability to process complex datasets and extract valuable information from large multidimensional detection data.^[^ ^35^, ^36^, ^37^ ^]^ Thus, developing a multimodal In situ analysis of EV‐circRNAs to combine ML algorithms not only improves the accuracy of biomarker detection but also offers great potential for explaining the complex roles of EV‐circRNAs in diseases.

**Scheme 1:**
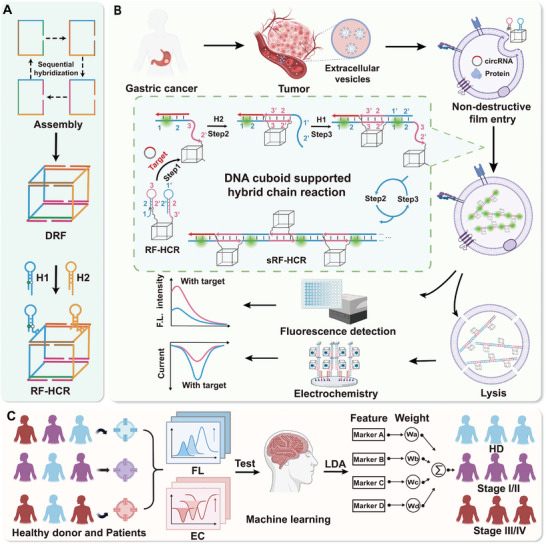
Flowchart of BEISA application in EV‐circRNA analysis. A) Schematic diagram of RF‐HCR assembly. B) Application of BEISA for in situ detection of EV‐circRNA. C) Data processing and machine learning analysis for cancer diagnosis.

In this study, we developed a bimodal EV‐circRNA in situ analyzer (BEISA) using a DNA rectangular frame‐guided hybridization chain reaction (RF‐HCR) system. By combining ML algorithms and bimodal EV‐circRNA in situ profiling, this platform enabled the diagnosis and staging of GC. The analysis process consisted of three parts (Scheme 1): RF‐HCR synthesis, bimodal analysis for EV‐circRNA profiling, and ML‐based cancer diagnosis. First, to enable direct in situ detection of circRNA without the need for EVs lysis or RNA extraction, we designed a compact DNA rectangular framework (DRF) and integrated the HCR component to form an RF‐HCR (Scheme 1A). The RF‐HCR could specifically recognize the target in its presence, leading to the disruption of the stable folded structure of H1. The site‐exposed H1 acted as a target for H2, which opened H2 and released a sequence identical to the initial circRNA. This triggered further HCRs, ultimately forming an ever‐extending RF‐HCR with a stranded arrangement (sRF‐HCR). This process achieved an efficient output of sRF‐HCR fluorescence signals by labeling the fluorophore 5‐FAM and quencher 3‐BHQ1 on the hairpin structures of H1 and H2, respectively. The released sRF‐HCR was enriched in the electrodes by perforating the EVs to achieve electrochemical signal sensing (Scheme 1B). Finally, in a clinical cohort of patients with GC, a bimodal signaling assay for four circRNAs was used in combination with ML techniques (Scheme 1C). This platform achieved 86.7% sensitivity, 90.0% specificity, 88.0% accuracy, and high efficiency, with an area under the curve (AUC) of 0.9250, effectively differentiating between HDs and patients with GC. It also achieved an 85.0% diagnostic sensitivity for early and progressive GC. We anticipate that the BEISA will be a promising tool for advancing the analysis of tumor‐derived EV‐circRNAs and improving the clinical indications of circRNA‐based liquid biopsies.

## 2 Result

### 2.1 In Vitro Feasibility of RF‐HCR‐Based Bimodal Platforms

**Figure 1:**
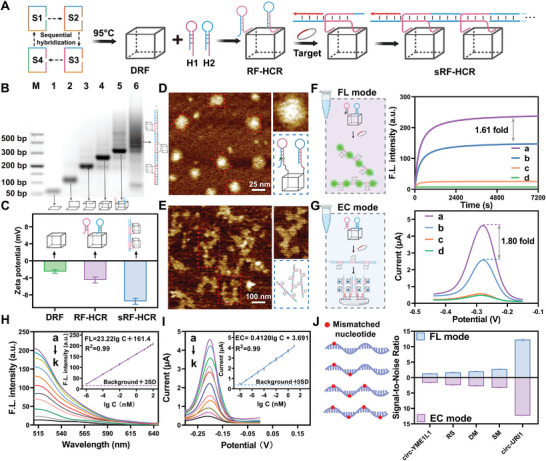
In vitro feasibility of RF‐HCR‐based bimodal platform. A) Schematic illustration of RF‐HCR preparation and sRF‐HCR formation via hybridization chain reaction (HCR). B) 2% agarose gel electrophoresis was used to characterize the assembly of RF‐HCR and sRF‐HCR. C) Construction of RF‐HCR and zeta potential analysis of sRF‐HCR. D) AFM imaging of RF‐HCR. Scale bar, 25 nm. E) AFM imaging of sRF‐HCR. Scale bar, 100 nm. F) Time‐dependent fluorescence response of RF‐HCR and traditional HCR in the presence of 50 nm circ‐URI1. The concentration of RF‐HCR and the nude hairpin is 100 nM. RF‐HCR a), traditional HCR b), RF‐HCR without target c), traditional HCR without target d). a.u., arbitrary units. G) DPV analysis of RF‐HCR and traditional HCR in the presence of 10 nm circ‐URI1. The concentration of RF‐HCR and naked hairpin was 20 nm. H) The fluorescence intensity from the addition of different concentrations of synthesized target based on RF‐HCR (from a to k: 50 nm, 25 nm, 10 nm, 1 nm, 100 pm, 10 pm, 1 pm, 100 fM, 10 fM, 1 fM, and 0 nM). The inset shows the calibration curve of fluorescence intensity versus the logarithm of the target. a.u., arbitrary units. I) DPV analysis from the addition of different concentrations of synthesized target based on RF‐HCR (from a to k: 10 nm, 1 nm, 100 pm, 10 pm, 1 pm, 100 fM, 10 fM, 1 fM, 100 aM, 10 aM, and 0 aM). The inset shows the calibration curve of the current versus the logarithm of the target. J) The signal‐to‐noise ratio of the target (circ‐URI1), single‐base mismatch sequence (SM), double‐base mismatch sequence (DM), three‐base mismatch sequence (RS), and a fully mismatched sample (circ‐YME1L1) at a concentration of 20 nm. Data represent mean ± SD (*n* = 3).

To illustrate the feasibility of the RF‐HCR‐based bimodal platform in vitro, we designed and constructed a circRNA‐triggered RF‐HCR system (**Figure** **1**A). Circ‐URI1 was chosen as the target because of its upregulation in plasma EVs of patients with GC.^[^ ^38^ ^]^ We successfully characterized the synthesized circ‐URI1 for use in subsequent experiments (Figure S1, Supporting Information). The RF‐HCR assembly process was characterized by agarose gel electrophoresis (Figure 1B). The successful formation of the DNA rectangular frameworks was characterized by agarose gel electrophoresis. As shown in Figure 1B, the sequences were combined into a single DNA rectangular framework (Lanes 1 to 4) in the form of one strand (S1), two strands (S1 + S2), three strands (S1 + S2 + S3), single DNA rectangular framework (S1 + S2 + S3 +S4). Then, the complete RF‐HCR (S1 + S2 + S3 + S4+ H1 + H2) was constructed based on a rectangular DNA framework, and as more DNA hairpins were assembled on the DRF, larger and higher‐purity bands were formed (Lane 5) (Figure S2, Supporting Information). The molecular weights of the bands increased further after co‐incubation with the target (lane 6), indicating the effectiveness of the HCR circuit of the RF‐HCR system. In addition, the zeta potential of the RF‐HCR was lower than that of the original rectangular DNA framework (Figure 1C), which was attributed to the negative electrical enhancement of the RF‐HCR assembly. When the target was present, the RF‐HCR triggered an efficient HCR to form a tandem sRF‐HCR, and the zeta potential distribution was further reduced from −4.5 to −9.5 mV. DLS further confirmed the successful construction of the RF‐HCR (Figure S3, Supporting Information). Atomic force microscopy demonstrated that the synthesized RF‐HCR exhibited a rectangular shape and good dispersion (Figure 1D), and the addition of the target resulted in a larger and well‐ordered tandem rectangular shape (Figure 1E). The superior properties of the RF‐HCR for circRNA detection were further verified using fluorescence and electrochemical tests. Compared with the conventional HCR, the RF‐HCR showed a 1.61‐ and 1.80‐fold enhancement in the FL and DPV signals, respectively, with little change in the background signal (Figure 1F,G). This significant signal enhancement was attributed to the spatial confinement effect of the DNA scaffold.^[^ ^39^, ^40^ ^]^ In addition, we confirmed the assembly of the sRF‐HCR on the electrode surface (Figure S4, Supporting Information), and demonstrated the high stability of sRF‐HCR at different concentrations of target circRNA by fluorescence kinetics monitoring (Figure S5, Supporting Information). The above experiments illustrated the successful synthesis of the RF‐HCR and good target responsiveness of the RF‐HCR‐based bimodal platform.

Under the optimal experimental conditions (Figure S6, Supporting Information), we constructed standard fluorescence and electrochemistry curves for different concentrations of circ‐URI1 using an RF‐HCR mixed with solutions containing a range of circ‐URI1 concentrations (Figure 1H,I). FL and DPV signals were positively correlated with the circ‐URI1 concentration, indicating that the bimodal sensors exhibited a robust linear relationship in response to increasing target concentrations. The linear regression fitting equations were FL = 23.22lgC + 161.4 (R^2^ = 0.99) and EC = 0.412lgC + 3.691 (R^2^ = 0.99). According to the calculation method proposed by the International Union of Pure and Applied Chemistry (IUPAC), the equation for the limit of detection (LOD) is as follows: LOD = blank signal+ + 3×standard deviation of the blank. The LODs were 0.55 fM and 8.70 aM, which were ≈890‐ and 792‐fold more sensitive than the conventional HCR reaction (Figure S7, Supporting Information), respectively. The RF‐HCR‐based bimodal platform exhibited good stability and reproducibility (Figure S8, Supporting Information). Using the target‐specific RF‐HCR system, the remarkable specificity of circ‐URI1 for discrimination of potential interfering substances was observed (Figure 1J; Figure S9, Supporting Information). Together, these results demonstrated that the dual‐mode assay was distinguished by its high sensitivity, low detection limit, and exceptional detection performance.

### 2.2 Feasibility of the BEISA for the In Situ Analysis of EVs

**Figure 2:**
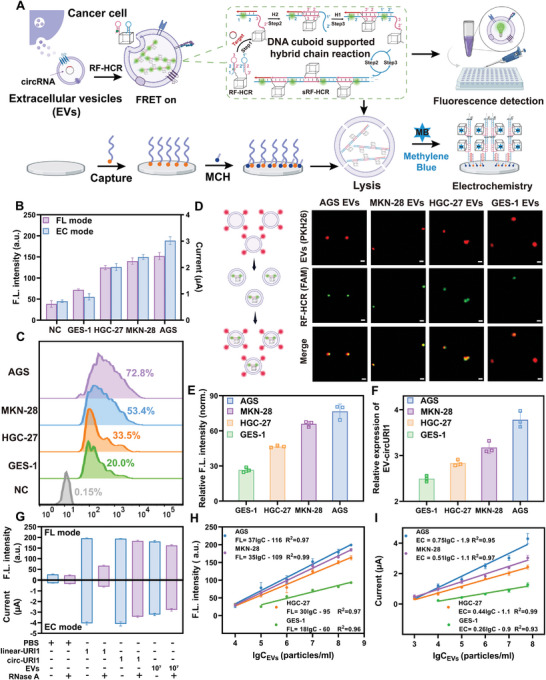
Feasibility of BEISA applied to in situ bimodal analysis of EVs. A) Schematic diagram of the BEISA construction process. B) Fluorescence and electrochemical intensity of circ‐URI1 expression in gastric cancer cell line‐derived EVs (10^7^ particles per mL) and normal cell‐derived EVs (10^7^ particles per mL) after incubation with RF‐HCR (100 nm). C) Nanoflow cytometry analysis of circ‐URI1 expression in tumor and normal cell‐derived EVs. RF‐HCR at a concentration of 10 nm and EVs at a concentration of 10^5^ particles per mL. D) Super‐resolution optical microscopy images of the tumor and normal cell‐derived EVs. PKH 26 staining of EV membranes (red, top), 515 nm fluorescence from FAM on RF‐HCR (green, middle), and co‐localized signals (bottom). Scale bar, 150 nm. RF‐HCR at a concentration of 10 nm and EVs at a concentration of 10^6^ particlesper mL. E) Quantitative analysis of FAM fluorescence intensity in different EVs. F) qRT‐PCR detection of EV‐circURI1 expression in different cell lines. G) Determination of synthetic linear‐URI1 or EV‐circURI1 with or without standard RNase A preparations. H) Linear correlation analysis between fluorescence signals and EVs concentration (3.1 × 10^8^, 10^8^, 10^7^, 10^6^, 10^5^, and 10^4^ particles per mL). I) Linear correlation analysis between electrochemical signal and EVs concentration (6.2 × 10^7^, 10^7^, 10^6^, 10^5^, 10^4^ and 10^3^ particles per mL). Data represent mean ± SD (*n *= 3).

Based on the superior in vitro detection performance of the RF‐HR system, we further explored a BEISA based on the RF‐HCR. The BEISA process included the key aspects of nondestructive RF‐HCR access, signal amplification, and bimodal detection (**Figure** **2**A and S10). We extracted EVs from three different GC cell lines (AGS, MKN‐28, and HGC‐27) and a normal human gastric mucosal epithelial cell line (GES‐1) using standard ultracentrifugation. The extracted EVs were characterized following a standard protocol. Electron microscopy revealed typical partially folded discs with a diameter of 100 nm (Figure S11A, Supporting Information). Western blot analysis (Figure S11B, Supporting Information) confirmed the specific protein labeling of EVs (positive proteins: CD9, CD63, and CD81). NTA showed that the particles of the four extracted EVs were ≈100–200 nm (Figure S12, Supporting Information). These results were consistent with the previous reports,^[^ ^41^, ^42^, ^43^ ^]^ indicating that the isolated EVs exhibited high levels of purity and integrity, rendering them suitable for subsequent experiments.

To verify the feasibility of the BEISA for the in situ analysis of EVs, we chose AGS cell‐line‐derived EVs as a model and used circ‐URI1 as a target, which was validated by substrate‐deletion experiments. The results showed that the BEISA could detect AGS‐derived EVs‐circ‐URI1 when all substrates were present (Figure S13, Supporting Information). To achieve the best analytical performance of in situ bimodal signal, we optimized the size of the RF and selected 34 bp as the optimal edge length for subsequent experiments (Figure S14, Supporting Information). Fluorescence signal analysis showed that the fluorescence intensities of tumor cell EVs, in descending order, were highest in AGS, followed by MKN‐28, and HGC‐27. All of these intensities were significantly higher than those of EVs from normal GES‐1 cells. The electrochemical signals remained highly consistent across the different cell lines (Figure 2B). In addition, nanoflow cytometry and super‐resolution microscopy (Figure 2C–E) further showed that AGS cells experienced the highest expression of EV‐cirURI1, followed by MKN‐28 cells and that the expression of EV‐cirURI1 was higher than that of the normal cell lines in all tumors. The accuracy of the BEISA was also demonstrated by quantitative RT‐qPCR (Figure 2F).

To confirm the source of the in situ bimodal signal, we first used RNase A to treat the synthesized linearURI1 and circ‐URI1. The results showed that the bimodal signal of linearURI1 was reduced to the negative control level after RNase A treatment. On the contrary, the signal of circ‐URI1 barely changed (Figure 2G). Then, we introduced RNase A into the AGS cell‐derived EVs by electroporation. The results exhibited that the bimodal signals of treated EVs were only slightly decreased compared with those of untreated EVs. These results suggested that the bimodal signal was triggered by the target circ‐URI1 rather than linearURI1. Furthermore, no significant increase in fluorescence was observed upon continuous monitoring of the supernatant for 4 h after incubating the RF‐HCR system with EVs. This confirmed that the entry process of the RF‐HCR probe into the membrane was non‐destructive and did not lead to nucleic acid leakage (Figure S15, Supporting Information). Moreover, compared with the RF‐HCR used in EVs lysis, the BESIA significantly reduced the loss of EV‐circRNA and showed higher sensitivity and a wider detection range (Figure S16, Supporting Information) with negligible loss of sRF‐HCR during EV lysis in electrochemical mode (Figure S17 and Table S2, Supporting Information).

Finally, we comprehensively evaluated the detection performance of the BEISA platform by analyzing the expression levels of circ‐URI1 in AGS‐, MKN‐28‐, HGC‐27‐, and GES‐1‐derived EVs. The results showed a strong linear relationship between the fluorescence and electrochemical signals and the concentration of EVs in each cell line, with correlation coefficients (R^2^) exceeding 0.90 (Figure 2H,I). These results highlighted the high accuracy and reliability of the BEISA platform for EV‐circRNA analysis. In addition, we explored the unique advantages of the RF‐HCR in in situ assays and compared them with a conventional hairpins. The results showed that the RF‐HCR exhibited significant advantages over conventional hairpins for EV‐circRNA analysis (Figures S18 and S19, Supporting Information).

In summary, we are confident of the universality and reliability of the BEISA platform for the in situ analysis of multimodal EV‐circRNAs. It offers great potential for expanding the use of EV‐circRNA analysis in clinical settings and is expected to facilitate the development of non‐invasive cancer diagnostic techniques.

### 2.3 Clinical Feasibility of the BEISA for EV‐circRNA Profiling

**Figure 3:**
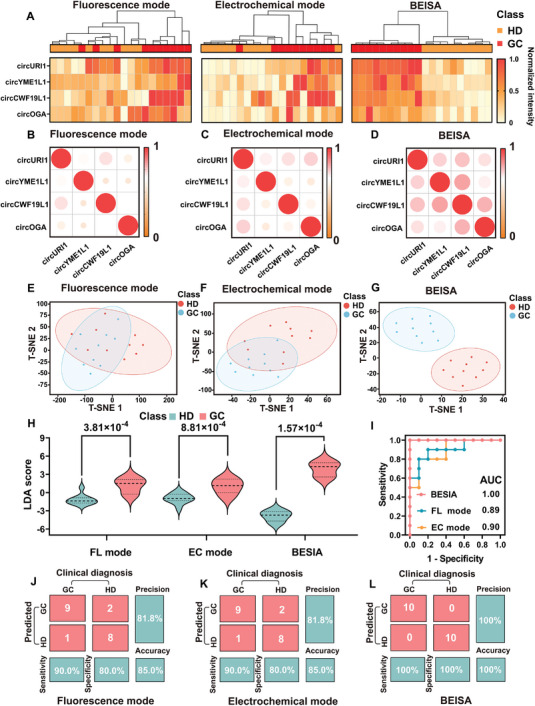
Clinical evaluation of BESIA for tumor‐derived EV‐circRNA profiling. A) Unsupervised hierarchical clustering (Pearson correlation, average linkage) heatmap of the expression levels of four circRNAs in fluorescence platform, electrochemical platform, and BEISA for differentiating between GC (*n *= 10) and HD controls (*n *= 10). Signal intensities of each circRNA were normalized using the min‐max normalization method. B–D) Correlation matrices of the expression profiles of the four circRNAs in the fluorescence platform B), electrochemical platform C), and BEISA D). E–G) t‐Distributed stochastic neighbor embedding (t‐SNE) differentiated between GC patients and HD controls using the four circRNAs in the fluorescence platform E), electrochemical platform F) and BEISA G) as input. (H) LDA scores of fluorescence platform, electrochemical platform, and BEISA for differentiating GC patients from HD controls. *p* values were determined by nonparametric, two‐tailed Mann–Whitney *U* test. I) ROC curves of fluorescence platform, electrochemical platform, and BEISA for differentiation between GC patients and HD controls. J–L) Confusion matrices for fluorescence platform, electrochemical platform, and BEISA. All statistical analyses were performed with 95% confidence intervals.

To preliminarily evaluate the potential clinical application of the BEISA in the diagnosis of GC, we collected plasma samples from ten patients (GC) and 10 HDs (Table S3, Supporting Information). Our objectives were to verify whether the BEISA could i) analyze tumor‐derived EVs in plasma samples and ii) improve the diagnostic performance of EV‐based liquid biopsies. In a preliminary study, we screened several EV‐circRNAs in our database that may have been upregulated in GC in our database, including EV‐circURI1, EV‐circYME1L1, EV‐circCWF19L1, and EV‐circOGA. Using the collected samples, we performed multiparametric circRNA analyses (weighted sums of bimodal platforms, with optimal weights determined by logistic regression) using the BEISA. For comparison, single fluorescence and electrochemical platforms for EV‐circRNA profiling were used in the clinical cohort (**Figure** **3**A).

We used an unsupervised hierarchical clustering approach to explore whether the four circRNAs from each patient showed mutually exclusive or similar expression patterns across different assay platforms. Expression heat maps revealed considerable heterogeneity in the abundance of each circRNA across the different assay platforms, and these heterogeneities showed significant differences in distinguishing an HD from a GC patient. The heterogeneous expression profiles of the circRNAs in these three assay systems could be categorized into two distinct unsupervised classes. All four circRNAs were upregulated in GC plasma samples compared with HD plasma samples. These comparative analyses revealed heterogeneity among different assay platforms and confirmed the validity of the analyzed data. Pairwise comparisons of the four circRNAs from the different platforms are shown (Figure 3B–D). The finding that these four circRNAs did not show a strong correlation among the three modalities prompted us to adopt a multi‐marker co‐detection approach to improve the diagnostic accuracy of GC. To assess the diagnostic ability of these three assay systems for GC, we used t‐distribution stochastic neighbor embedding to distinguish GC patients from HDs (Figure 3E–G). The BEISA demonstrated a smaller sample overlap, indicating its superiority in distinguishing between the two groups of samples. To further improve the diagnostic performance of EV‐based liquid biopsy in distinguishing between HD and GC groups, we conducted receiver operating characteristic (ROC) analysis to assess the diagnostic efficacy of each circRNA (Figure S20, Supporting Information). However, the diagnostic performance of a single marker failed to reach the desired level, which prompted us to use a multiparameter combination combined with ML to enhance the diagnostic accuracy. All circRNA profiles were integrated using linear discriminant analysis (LDA), which was then applied to plot ROC curves for the three detection platforms. The results showed (Figure 3H) that the difference in LDA values for the BEISA was more statistically significant (*p *= 1.57 × 10^−4^) compared with those for the single fluorescence (nonparametric two‐tailed Mann–Whitney *U* test, *p *= 3.81 × 10^−4^) and electrochemical platforms (*p *= 8.81 × 10^−4^). In addition, the AUC of the BEISA was 1.00, which far exceeded those of the single fluorescence and electrochemical platforms (Figure 3I). The classification results from the confusion matrix further confirmed that the BEISA demonstrated superior sensitivity, specificity, and accuracy (each reaching 100%) for the differential diagnosis of GC compared with those of the single assay platforms (Figure 3J–L). We further established an independent cohort (*n *= 15) to test the samples with our platform (Figure S21 and Table S3, Supporting Information). The BESIA with dual mode also obtained a higher accuracy (93.3%) than that of single mode (86.7% and 80.0%).

In summary, the BEISA provided a highly accurate, sensitive, and specific liquid biopsy method for GC diagnosis, outperforming conventional unimodal sensors and accurately classifying tumor‐derived EVs in plasma samples.

### 2.4 Training Cohort Study of BEISA‐Based EV‐circRNA Profiling in GC Diagnosis

To assess the diagnostic suitability of the BEISA, plasma was collected from 75 patients participating in a clinical cohort (Table S4, Supporting Information), which included HDs (*n *= 25), patients with stage I–II disease (*n *= 25), and patients with stage III–IV disease (*n *= 25). From these samples, 45 out of the 75 plasma samples were selected by stratified random sampling to construct a training cohort. Using specific circRNA markers, the training cohort was analyzed to generate a discriminant function model through LDA. Then, this model was used to classify and diagnose patients in the validation cohort.

**Figure 4:**
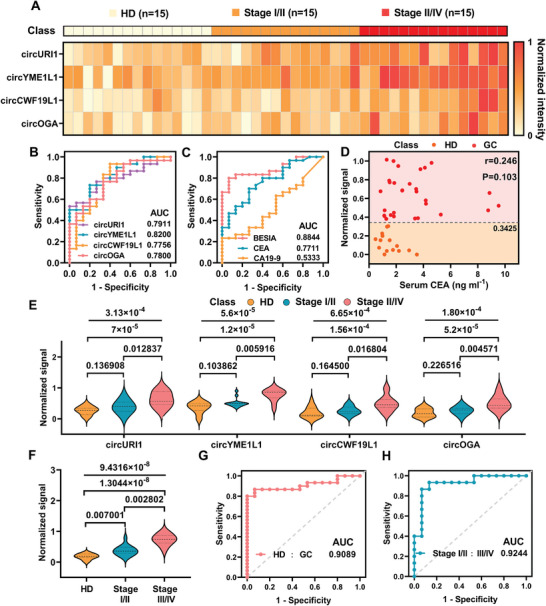
BEISA distinguishing HD, stage I–II, and stage III–IV in the training cohort. A) Heatmap showing the relative abundance of the four circRNAs in the training set of HD (*n *= 15), stage I‐II (*n *= 15), and stage III‐IV (*n *= 15) patients. Signal intensities of each circRNA were normalized using the min‐max normalization method. B,C) ROC curves for individual markers B) and BEISA signals used to diagnose GC C). D) Correlation of BEISA signals with serum CEA to differentiate HD from GC in the training cohort. The dashed line indicates the threshold value for positivity (BEISA signal, 0.3425). E,F) Expression levels of single circRNA markers E) and combined markers F) detected by BEISA. The Kruskal–Wallis one‐way ANOVA was used to determine overall and group pair *p* values. G) ROC curves were plotted to identify GC patients using the LDA results in the training cohort. H) ROC curves for diagnosing early gastric cancer (I‐II) were plotted using the LDA results from the validation cohort. The overall diagnostic accuracy of the BEISA signal was 86.7%. All statistical analyses were done at 95% confidence intervals, ensuring the reliability and validity of the results.

For the analysis of the training cohort, plasma samples were collected from 15 HDs, 15 patients with stage I–II GC (stage I–II), and 15 patients with stage III–IV GC (stage III–IV). The expression levels of the four circRNAs were quantified in each participant in the training cohort (**Figure** **4**A). The results of BEISA showed that these circRNAs exhibited significant heterogeneity in distinguishing between the HD and GC patient groups. Diagnostic indicators for individual markers or marker combinations were assessed using ROC curve analysis (Figure 4B,C). None of the four circRNA markers showed sufficiently high sensitivity, specificity, or accuracy. The four‐marker profile (AUC = 0.8844, sensitivity = 80.0%, specificity = 90.0%, and accuracy = 82.5%) provided better diagnostic capabilities than the individual markers in the training cohort.

In addition, the correlation between BEISA signals and serum carcinoembryonic antigen (CEA) and glycan antigen 19‐9 (CA19‐9) in patients with GC were investigated (Figure 4D; Figure S22, Supporting Information). In the training set, the BEISA signal showed no significant correlation with either CEA and CA19‐9 (r_CEA_ = 0.246, P_CEA_ = 0.103; r_CA19‐9_ = 0.087, P_CA19‐9_ = 0.572), demonstrating that the platform provides a diagnostic method independent of traditional tumor markers and is able to enhance the accuracy and specificity of GC detection. Furthermore, we evaluated the efficacy of the assay in differentiating stage I–II patients from stage III‐IV patients (Figure 4E,F). The results showed that EV‐circRNA profiling significantly improved the accuracy of patient grouping compared to individual markers (Kruskal–Wallis test, *p *= 9.4316 × 10^−8^). To further characterize the validity of BEISA for GC diagnosis, we plotted an LDA‐based ROC curve using ML (Figure 4G). The results showed that for the differentiation of HD and GC in the training cohort, BEISA had a high sensitivity of 86.7%, specificity of 90.0%, and accuracy of 87.5%, with an AUC of 0.9089. For the differentiation of stage I–II and stage III–IV patients in the training cohort, the overall accuracy was 86.7%, with an AUC of 0.9244 (Figure 4H).

### 2.5 Validation Cohort Study of BEISA‐Based EV‐circRNA Profiling in GC Diagnosis

**Figure 5:**
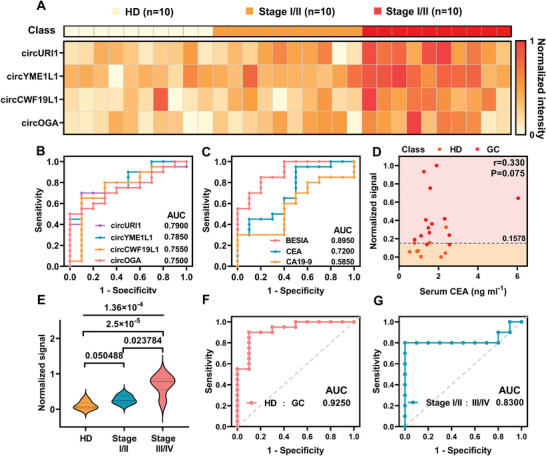
BEISA distinguishing HD, stage I‐II, and stage III‐IV in the validation cohort. A) Heatmap presenting the expression abundance of specific circRNAs in an age‐matched, independent validation cohort comprising 10 cases of HD, 10 cases of stage I‐II, and 10 cases of stage III‐IV. Data processing methods were similar to those employed in the training cohort (Figure 4A). B,C) ROC curves of single circRNAs and circRNAs profiles were used to discriminate HD from GC in the validation cohort. D) Correlation of BEISA signals with serum CEA to differentiate HD from GC in the validation cohort. E) BEISA to detect the expression level of circRNA profiles. Overall group pair *p* values were calculated similarly to those in the training cohort. F) ROC curves of identified GC patients were plotted using the LDA results in the validation cohort. G) ROC curves for diagnosing early gastric cancer (I‐IIA) were plotted using the LDA results from the validation cohort. The overall diagnostic accuracy of the BEISA signal was 85.0%. All statistical analyses were done at 95% confidence intervals, ensuring the reliability and validity of the results.

In this study, the classification method was further applied to an independent validation cohort consisting of plasma samples from 30 age‐matched subjects, including 10 HDs, 10 patients with stage I–II GC, and 10 patients with stage III–IV GC. The expression levels of the four circRNAs are summarized for each subject in the validation cohort (**Figure** **5**A). The results of the analysis of the marker expression heatmap obtained using the BEISA re‐emphasized the significant heterogeneity in distinguishing HDs from GC patients. In all validation cohorts (Figure 5B,C), the BEISA signal demonstrated excellent diagnostic performance, achieving an AUC of 0.8950, with sensitivity, specificity, and accuracy all at 80.0%, outperforming single‐marker approaches. In addition, the study explored the correlation between the BEISA signal and serum levels of CEA and CA19‐9 in patients with GC (Figure 5D; Figure S23, Supporting Information). The results showed no significant correlation between the BEISA signal and either CEA or CA19‐9 levels (r_CEA_ = 0.330, P_CEA_ = 0.075; r_CA19‐9_ = 0.157, P_CA19‐9_ = 0.408), highlighting that the platform provided a diagnostic strategy independent of traditional tumor markers and enhanced the accuracy and specificity of GC detection.

To assess the efficacy of the assay in differentiating between HDs and patients with stages I–II and III–IV GC, the validation cohort data was analyzed using the Kruskal–Wallis test. The results (Figure 5E; Figure S24, Supporting Information) showed that the combination of circRNA markers (*p *= 1.36 × 10^−4^) significantly improved the differentiation between the three subject groups compared with the use of single markers. Subsequently, the validation cohort data was inputted into the previously trained ML model to assess its effectiveness in cancer diagnosis. The diagnostic efficacy of the BESIA combined with ML in patients with stage I–II and III–IV GC was further revealed by the ROC plots obtained using LDA. In the validation cohort, the BEISA signal differentiated between HDs and patients with GC with 86.7% sensitivity, 90.0% specificity, and 88.0% accuracy (AUC = 0.9250) (Figure 5F). For the diagnosis of early‐stage GC, the overall accuracy still reached 85.0% with an AUC of 0.8300, despite the presence of a few (3 of 10) misclassification cases (Figure 5G). Taken together, these results further demonstrated that the RF‐HCR‐based BEISA could be used for the clinical diagnosis of EV‐circRNAs and to improve the diagnostic performance for early‐stage cancers.

## 3 Discussion

In the past decade, EVs as targets in tumor liquid biopsies have garnered significant research interest, leading to a surge in related studies. In recent years, EV‐circRNA in circulating blood has been recognized as a highly promising biomarker for cancer diagnosis because of its differential expression in early‐stage cancer patients compared with that in the healthy population. However, the detection and analysis of EV‐circRNAs face many challenges, including species diversity, the complexity of mechanisms of action, significant heterogeneity of expression patterns among patients, need for low‐abundance assays in early disease stages, and maintenance of stability and reliability during sample processing. Collectively, these challenges pose a dilemma in resolving EV‐circRNA information, limiting the realization of its value for clinical applications.

In this study, we developed a BEISA using a DNA RF‐HCR system. The platform offers several significant advantages: it eliminates the need for EV lysis and RNA extraction, which simplifies experimental operations and reduces sample loss. It also adopts a fast and stable signal amplification strategy that combines fluorescence and electrochemical detection systems to achieve dual signal output, which improves the accuracy and sensitivity of the assay. The platform also revealed different EV‐circRNA expression profiles in the plasma samples from HDs and patients with GC. These differences may not be fully revealed by traditional unimodal detection methods. The application of bimodal detection technology provides a richer and more comprehensive dimension for data collection, which significantly enhances the depth and breadth of subsequent analyses and interpretations.

ML technology has demonstrated excellent capabilities in handling complex biomedical datasets. The BEISA platform combines multimodal detection with advanced ML algorithms to differentiate between HDs and patients with GC with 86.7% sensitivity, 90.0% specificity, and 88.0% accuracy (AUC = 0.9250). More importantly, in distinguishing different clinical stages of GC, the BEISA platform combined bimodal detection with ML technology to achieve an overall accuracy of 85.0%. Compared with traditional GC diagnostic methods, the platform proposed in this study not only significantly improves diagnostic accuracy but also provides an important reference for individualized treatment. We also demonstrated a standard procedure for clinical sample pretreatment and ML analysis to ensure the repeatability and comparability of results in clinical samples, which facilitates the clinical transformation of EV detection technology, and accelerates the clinical application of EV‐RNAs.

As the next step, we are committed to expanding the research scope of EV‐circRNA markers and screening and validating additional biomarker combinations to improve the clinical detection accuracy of the BEISA platform. With the expansion of the study scale, we will validate the generalizability and stability of the BEISA using more patient samples. Meanwhile, the rich data accumulated will facilitate further development of our ML algorithms with a view to achieving more accurate multi‐marker analysis. We believe that these efforts will promote the widespread adoption of the BEISA in clinical applications and provide more accurate diagnostic tools for patients.

## 4 Experimental Section

### Ethical Statement and Study Participant Details

All clinical plasma samples used in this study were obtained from Southern Medical University Nanfang Hospital and strictly adhered to the ethical guidelines set out in the 1964 Declaration of Helsinki. The study was formally approved by the Ethics Committee of Southern Medical University Nanfang Hospital (Approval No. NFEC‐2022‐220). All samples (110 cases in total) were collected anonymously before treatment, and relevant information such as age and pathological diagnosis of the participants was recorded. The sample composition was as follows: a discovery cohort for initial validation of the efficacy of the four EV‐circRNAs in gastric cancer diagnosis and a training and validation cohort for machine learning model development. Specifically, the discovery cohort consisted of 15 healthy control individuals and 20 pretreatment gastric cancer patients; the training set contained 15 healthy control individuals, 15 patients with early gastric cancer, and 15 patients with advanced gastric cancer; and the testing set consisted of ten healthy control individuals, ten patients with early gastric cancer, and ten patients with advanced gastric cancer. For more information on clinical trial participants, see Tables S3 and S4 (Supporting Information).

### Materials and Instruments

The oligonucleotides utilized in this study were synthesized and subsequently purified via polyacrylamide gel electrophoresis (PAGE) at the Beijing Genomics Institution. The sequence details are provided in Table S1 (Supporting Information). The 4S Red Plus nucleic acid stain was procured from Sangon Inc. The 1× TE buffer was obtained from Biosharp. The TNaK buffer was configured in the laboratory, consisting of 20 mm Tris‐HCl, 20 mm KCl, and 125 mm NaCl (pH 7.5) for hybridization. Gibco, Thermo Fisher Scientific (MA, USA) provided essential media and buffers, including Phosphate Buffered Saline (PBS) and Roswell Park Memorial Institute 1640 (RPMI‐1640). The cell cultures and fetal bovine serum (FBS) were obtained from Procell (Wuhan, China). The electrochemical reagents utilized in the present study were 6‐mercapto‐1‐hexanol (MCH) and RuHex, both of which were obtained from Sigma‐Aldrich (St. Louis, Missouri, USA).

### Preparation of RF‐HCR

First, S1, S2, S3, and S4 were mixed in the ratio of 1:1:1:1 and diluted appropriately with TNAK buffer. The DNA rectangular framework was prepared by heating at 95 °C for 5 min using a PCR instrument and then slowly cooled to room temperature. The DNA rectangular (100 µm) framework and two hairpins (100 µm) were then incubated at 25 °C for 60 min to prepare RF‐HCR by sticky‐end hybridization. The RF‐HCR solution was stored at 4 °C, and protected from light.

### Nucleic Acid Electrophoresis

Agarose gel electrophoresis verified the assembly of RF‐HCR and sRF‐HCR presence. DNA products were electrophoresed in 0.5× TBE buffer (1 EDTA, 45 mm Tris‐boric acid, pH 8.0) at 140 V for 45 min. Imaging utilized a Sygene Gel imaging system.

### Atomic Force Microscopy (AFM) Imaging

AFM imaging was conducted with 50 nm RF‐HCR and 20 nm sRF‐HCR. Samples were placed on a positively charged mica plate for 15 min, followed by two rinses with ultrapure water. Imaging was performed in tapping mode using a Dimension 3100 AFM (Veeco, Germany). The image background was flattened using Nanoscope IIIa software.

### Preparation of Electrochemical Biosensor

First, the 2 mm gold disc electrode was polished with aluminum oxide powder for 6 min. Then, the electrode was cleaned with an ultrasonic cleaner and distilled water until no turbidity was observed. The bare gold electrodes were cleaned three times with piranha solution (H_2_O_2_ = 1:3) for 10 min each time. After rinsing with PBS to remove impurities, the capture probe was added dropwise to the gold electrode surface and left overnight at 4 °C for the gold‐thiol reaction.

### Electrochemical Analysis of BEISA

The gold electrode with the capture probe was incubated with MCH for 1 h to ensure the probe was as perpendicular to the gold surface as possible. RF‐HCR was mixed with the test sample and applied dropwise to the gold electrode for 30 min. DPV was performed in PBS buffer using a platinum wire electrode, a reference electrode, a modified gold electrode, and a test electrode. The buffer range was −0.5 to 0.2 V, with an amplitude of 0.05 V and a pulse width of 0.05 s.

### Fluorescence Measurements

CircRNA and EV‐circRNA were quantified using 100 nM RF‐HCR in a 60 µL luminescence spectrometer. The excitation wavelength was 490 nm at a constant voltage of 800 V, with a 10 nm gap between 510 and 650 nm. Fluorescence intensity was analyzed at 515 nm.

### Performance of BESIA

LOD is based on the smallest amount that can be confidently detected in a given analytical procedure. In this work, the LOD was calculated according to the International Union of Pure and Applied Chemistry recommendation (IUPAC, 1976), and the analyte signal at the limit of detection is given by the following equation:

(1) $$Y_{\mathrm{LOD}}=Y_{\mathrm{blank}}+k\times SD_{\mathrm{blank}}$$

WhereY__blank_ is the blank signal and SD_blank_ is the known standard deviation of the blank signal. According to Long and Winefordner (1983), the use of k = 3 allows a confidence level of 99.86% in the normal distribution of the blank signal to be used in the calculation of the signal response value of Y_LOD_. Then, based on the above data, equations, and calibration curves, the concentration X_LOD_ corresponding to Y_LOD_ can be found by inverse calculation.

### Cell Culture and Preparation

Human gastric cancer cells (AGS, MKN‐28, HGC‐27) were used as positive cells, while normal human gastric gland cells (GES‐1) served as control cells. All cells were obtained from Procell Life Technologies Ltd. with short tandem repeat identification reports. AGS, MKN‐28, and GES‐1 cells were cultured in RPMI‐1640 medium with 10% fetal bovine serum, whereas HGC‐27 cells had an additional 10% fetal bovine serum. All cells were incubated with a 1% penicillin‐streptomycin‐gentamicin mixture at 37 °C in a 5.0% CO2 atmosphere.

### Extraction of EVs

After the cells reach 80% fusion and are cultured in non‐EV serum for 12 h, EVs are isolated from the cells by ultracentrifugation of the culture supernatant at 300 g for 10 min, 2000 g for 20 min, and 10 000 g for 30 min to remove dead cells and cellular debris from the sediment. The supernatant was centrifuged twice at 135 000 g for 70 min in ultracentrifugation. Finally, suspend the EVs sediment in 100 µL of PBS. EVs were extracted from plasma using EV Separation Reagent (Ribobio, C10110‐2) according to the instructions. The 1 mL of plasma was separated by centrifugation of the collected donor blood samples at 300 g for 15 min. The plasma was then separated from the plasma by centrifugation at 2000 and 10 000 g for 20 min each. Transfer the plasma sample to a new tube, add a one‐third volume of EV Separation Reagent, and place the plasma mixture in the refrigerator for 30 min. Finally, the mixture is centrifuged at 15 000 g for 2 min and resuspended in 300 µL of PBS.

### Western Blot

After measuring cellular and EV protein concentrations using the BCA assay kit, proteins were separated on a 10% polyacrylamide gel (120 V, 60 min) and transferred to a PVDF membrane (200 mA, 60 min). Membranes were blocked with 5% fetal bovine serum before incubating with rabbit anti‐goat CD9, CD81, and TSG101 antibodies for 12 h. The membrane was then incubated with an enzyme‐labeled secondary antibody for 1 h. Immunoreactive bands were visualized using the FluorChem E system after reacting with the ECL reagent.

### qRT‐PCR Analysis

Total RNA was extracted from AGS, MKN‐28, HGC‐27, and GES‐1 cells using the Trizol kit. cDNA was synthesized using the SureScript First‐Strand cDNA Synthesis Kit. PCR amplification was conducted with specific primers designed online. Quantitative PCR was performed using the BlazeTaq SYBR Green qPCR Mix 2.0 kit on a CFX96 real‐time system. Reverse transcription to cDNA was done at 37 °C for 60 min and 95 °C for 5 min. cDNA amplification was detected with the SLAN96S system from Shanghai Hongshi Medical Technology Co. PCR conditions were 95 °C for 10 min, followed by 40 cycles of 95 °C for 10 s, 60 °C for 20 s, 72 °C for 10 s, and incubation at 37 °C.

### nFCM Analysis

EV was diluted to ≈10^6^ mL^−1^ with TNAK, then RF‐HCR was added to the EV‐containing buffer, and the final concentration of RF‐HCR was maintained at 100 nm. The above mixture was then incubated at 25 °C for 2.5 h, and unbound RF‐HCR was removed by ultrafiltration three times using an ultrafiltration tube. Finally, the samples were divided according to the instructions of the instrument (N30 Nanoflow Analyzer; NanoFCM, Inc.).

### Super‐Resolution Microscopy

A confocal disk was used to cover 50 µL of 1 mg mL^−1^ polylysine for 30 min. AGS cell‐derived EVs (6 × 10^7^ particles per µL) were incubated with 100 nm RF‐HCR (excitation wavelength 488 nm, emission wavelength 515 nm) for 2.5 h at room temperature. Subsequently, 0.25 µL of PHK26 Membrane Dye (excitation wavelength 551 nm, emission wavelength 567 nm) and 50 µL of dilution solution were added to the system for 30 min. The mixture was placed in a PLL confocal petri dish for 30 min and rinsed three times with PBS. Finally, images were captured using a super‐resolution microscope SIM system.

### Unsupervised Hierarchical Clustering Heat Map Analysis

The hierarchical clustering heatmap of Figure 3A is based on the fluorescence and electrochemical platforms after detecting the signals of four cyclic RNAs (EV‐circURI1, EV‐circYME1L1, EV‐circCWF19L1, and EV‐circOGA), respectively, and then calculating the optimal weights of the two signals through the logistic regression model and generating the bimodal signals by using a weighted sum method, and then Min‐Max Normalization was performed on the fluorescence, electrochemical, and bimodal signals, respectively, to normalize all the data to between 0 and 1 to eliminate the magnitude difference between the platforms. Subsequently, unsupervised hierarchical cluster analysis was performed on the four RNA signals from the three platforms using Pearson's correlation coefficient as a distance metric and combined with the average chaining method to generate a hierarchical clustering heat map. The heatmap demonstrated the signal intensity of different RNAs through color shades and clearly reflected the similarities and differences among samples through clustering. Compared with unimodal signals, bimodal signals (BEISA) show a stronger ability to group similar groups together, fully reflecting the importance of multidimensional data and the advantages of bimodal detection.

### Statistical Analysis

Mean, standard deviation (SD), and limit of detection (LOD) were calculated using standard formulas. Significance tests were performed using a two‐tailed Student's *t*‐test. The intensities of individual circRNA markers detected by the BESIA method were normalized using the minimum‐maximum (min‐max) approach, ensuring values fell between 0 and 1, as represented by the formula: X′ = (X – X_min_) / (X_max_ – X_min_). Hierarchical clustering of the analyzed markers was performed using a plug‐in for Origin 2021. t‐SNE analysis used four markers as inputs for binary classification (healthy donors and gastric cancer). The diagnostic performance of BESIA was calculated as the weighted sum of the normalized intensities of the four circRNA markers. For binary classification, *p* values for pairwise comparisons were determined using a nonparametric two‐tailed Mann–Whitney *U* test. For ternary classification, the Kruskal–Wallis one‐way ANOVA was used to determine overall and pairwise *p* values, with post hoc Dunn's test applied for pairwise multiple comparisons. ROC analyses were conducted on individual markers or combinations of markers to assess the area under the curve (AUC), sensitivity, specificity, and accuracy of a cancer diagnosis. The discovery cohort (*n *= 35) for validation of four EV‐circRNA in gastric cancer diagnosis. The training cohort (*n *= 45) was first analyzed to generate discriminant function models used to classify patients in the validation cohort (*n *= 30). Based on the training cohort, the optimal cut‐off point was selected using Youden's index and applied to assess the sensitivity, specificity, and accuracy of the validation cohort. All statistical analyses were performed with a 95% confidence interval (*p* < 0.05) using Origin 2021, GraphPad Prism (version 9.5.1), and SPSS 27.

### Machine Learning Analysis

In this study, linear discriminant analysis (LDA) was used as the primary machine learning algorithm to explore biomarker differences between cancer types. LDA is a classical supervised learning technique that maximizes between‐class differences while minimizing within‐class differences, enabling effective data reduction and classification. The signals of four EV‐circRNAs were detected based on an FL platform and an EC platform, respectively, and computed the optimal weights of FL and EC signals by logistic regression modeling to obtain a bimodal for each EV‐circRNA signal. Subsequently, the bimodal signals of these four EV‐circRNAs were input into the LDA model as features. The accurate classification of HDs, and GC patients, and patients with different stages of GC was finally realized.

## Conflict of Interest

The authors declare no conflict of interest.

## Supporting information



Supporting Information

## Data Availability

Research data are not shared.
