# Supplementary material for: Bimodal In Situ Analyzer for Circular RNA in Extracellular Vesicles Combined with Machine Learning for Accurate Gastric Cancer Detection
Source: Adv Sci (Weinh). 2025 Jan 17;12(15):2409202. doi: 10.1002/advs.202409202 (PMC12005762; doi:10.1002/advs.202409202)
Supplement: Supplementary file 1 — Supporting Information [file ADVS-12-2409202-s001.docx]

Supporting Information for

**Bimodal *In-Situ* Analyzer for Circular RNA in Extracellular Vesicles Combined with Machine Learning for Accurate Gastric Cancer Detection**

Yuhang Guo, Shihua Luo, Sinian Liu, Chao Yang, Weifeng Lv, Yuxin Liang, Tingting Ji, Wenbin Li, Chunchen Liu, Xin Li, Lei Zheng*, Ye Zhang*

**Keywords:** DNA nanotechnology, Bi-modal detection, circular RNA, early cancer diagnostics, machine learning

Y. Guo, S. Liu, C. Yang, Y. Liang, T. Ji, W. Li, C. L, X. L, L. Zheng, Y. Zhang

Department of Laboratory Medicine

Guangdong Engineering and Technology Research Center for Rapid Diagnostic Biosensors

Nanfang Hospital

School of Laboratory Medicine and Biotechnology

Southern Medical University

Guangzhou 510515, P.R. China

E-mail: nfyyzhenglei@smu.edu.cn; zhangye232@i.smu.edu.cn

W. Lv

Department of Laboratory Medicine

Foshan Traditional Chinese Medicine Hospital

Foshan 528000, P.R. China

S. Luo

Center for Clinical Laboratory Diagnosis and Research

Key Laboratory of Research on Clinical Molecular Diagnosis for High Incidence Diseases in Western Guangxi of Guangxi Higher Education Institutions

The Affiliated Hospital of Youjiang Medical University for Nationalities

Baise 533000, Guangxi, P.R. China

Y. Guo, S. Luo and S. Liu contributed equally to this work.

**1. Supplementary figures.**

**Figure S1.** Synthesis and characterization of circ-URI1. (A) Schematic diagram of circRNA synthesis. (B) 2% agarose gel electrophoresis to analyze circ-URI1 synthesis. Lane M, DNA ladder; Lane 1, linearURI1; Lane 2, linearURI1 + guide RNA + T4 RNA ligase 2; Lane 3, linearURI1 + guide RNA + RNase R; Lane 4, linearURI1 + guide RNA + T4 RNA ligase 2 + RNase R. (C) Fluorescence intensity response of RF-HCR in the presence of 50 nM circ-URI1, LinearURI1.

**Figure S2.** Hairpin structure designed by the Nupack platform for triggering circ-URI1 (green spots are adenine; blue spots are cytosine; black spots are guanine; red spots are thymine).

**Figure S3.** (A) DLS measurement of the particle size of DRF. (B) DLS measurement of the particle size of RF-HCR. (C) DLS measurement of particle size of sRF-HCR.

**Figure S4.** (A) EIS，(B) SWV and (C) CV of the bare gold electrode (a); the electrode modified with capture probe (b); the modified electrode after MCH block (c); sRF-HCR captured by the prepared electrode (d).

**Figure S5.** Time-dependent fluorescence response plots with different concentrations (50 nM, 0.5 nM, 50 fM and 0 nM) of the target (circ-URI1).

**Figure S6.** (A) Effect of reaction time of fluorescence and electrochemical platforms. (B) Effect of reaction temperature of fluorescence and electrochemical platforms. Data represent mean ± SD (*n*=3).

**Figure S7.** (A) In vitro performance evaluation of traditional HCR. The fluorescence intensity from the addition of different concentrations of synthesized target based on traditional HCR (from a to g: 50 nM, 25 nM, 10 nM, 1 nM, 100 pM, 10 pM and 1 pM). The inset shows the calibration curve of fluorescence intensity versus the logarithm of the target. a.u., arbitrary units. The LOD was 491 fM. (B) DPV analysis from the addition of different concentrations of synthesized target based on traditional HCR (from a to k: 10 nM, 1 nM, 100 pM, 10 pM, 1 pM, 100 fM and 10 fM). The inset shows the calibration curve of current versus the logarithm of the target. The LOD was 6.89 fM. Data represent mean±SD (*n*=3). (C) Concentration and regions of traditional HCR and RF-HCR. The outstanding sensitivity of RF-HCR was confirmed based on the collision theory (V = 1/*cN*), where *N* represents Avogadro's constant, *c* represents the reactant concentration, and V represents the localized sphere volume. When the solution of traditional HCR was 100 nM H1 and H2, the volume of the collision space was calculated to be 1.7×10^-17^ L. The combination of HCR with the DNA rectangular framework (RF-HCR) shortened the distance between H1 and H2 to 11.56 nm (34 bp), increasing the localized concentration of both H1 and H2 to 2053 μM.

**Figure S8.** Stability validation of fluorescence platform (A) and electrochemical platform (B). Assays were performed at week 0, week 1, week 2, week 3 and week 4. Data represent mean ± SD (*n*=3).

**Figure S9.** Heatmap analysis of four EV-circRNA orthogonal identifications on fluorescence platform (A) and electrochemical platform (B). Data represent mean ± SD (*n*=3).

**Figure S10.** Schematic diagram of the electrochemical biosensor EV-circRNA assay.

**Figure S11.** (A) TEM images of model EVS isolated from AGS cell lines. Scale bar, 50 nm. (B) Western blotting analysis of representative proteins of EVs obtained from AGS cells line.

**Figure S12.** Size distribution and concentration of EVs obtained from AGS (A), MKN-28 (B), HGC-27 (C), and GES-1 (D) cell lines by nanoparticle tracking analysis (NTA).

**Figure S13.** (A) Reaction substrate deletion experiments in fluorescence platform. (B) Reaction substrate deletion experiments in electrochemical platform. Data represent mean ± SD (*n*=3).

**Figure S14.** The effects of RF-HCR of different sizes on membrane entry efficiency (A), fluorescence spectrum (B), and electrochemical analysis (C). The numbers at the top of the columns indicate the signal-to-noise ratio.

**Figure S15.** The blue bar indicates the fluorescence intensity of the supernatant after RF-HCR penetration, and the purple bar indicates the fluorescence intensity of the supernatant of the original sample (without RF-HCR penetration). No significant increase in fluorescence intensity was observed even after 4 h, suggesting that RF-HCR-mediated delivery does not cause leakage of sEV cellular endosomes. *p* values were determined by a nonparametric, two-tailed Mann-Whitney *U* test. Data represent mean ± SD (*n*=3).

**Figure S16.** (A) Linear correlation analysis of RF-HCR *in-situ* and in EV lysis between fluorescence signals and EVs concentration, and between electrochemical signals and EVs concentration (B). Data represent mean ± SD (*n*=3).

**Figure S17.** Loss assessment of sRF-HCR in different concentrations of AGS-derived EVs in electrochemical mode (A-D). The *p* values were determined by a nonparametric two-tailed Mann-Whitney *U* test. Data represent mean ± SD (*n*=3).

**Figure S18.** Super-resolution optical microscopy images of EVs. RF-HCR/H1+H2 FAM fluorescence at 515 nm (green, left), EV membranes stained by PKH 26 (red, middle), and merged images (right). The scale bar is 5μm.To explore the unique advantages of RF-HCR (A) in accessing EVs, compared it with conventional hairpin-into-the-membrane methods, including electroporation (B) and standalone hairpin structure (C).

**Figure S19.** In vivo performance analysis of RF-HCR (A), electroporation (B). Concentrations of RF-HCR, H1, and H2 were all 100 nM. Insets show calibration curves of bi-modal signals versus the logarithm of the concentration of the EVs. Data represent mean ± SD (*n*=3).

**Figure S20.** ROC curves of individual EV-circRNA distinguishing GC and HD in a single fluorescence platform (A) and an electrochemical detection platform (B).

**Figure S21.** (A) Heatmap showing the relative abundance of the four circRNAs in the discovery set of HD (*n*=5) and GC patients (*n*=10). Signal intensities of each circRNA were normalized using the min-max normalization method. (B to D) ROC curves of fluorescence platform (B), electrochemical platform (C) and BEISA (D) for differentiation between GC patients and HD controls. (E to G) Confusion matrices for fluorescence platform (E), electrochemical platform (F), and BEISA (G).

**Figure S22.** Correlation of BEISA signals with serum CA19-9 in the training cohort to distinguish between HD and GC.

**Figure S23.** Correlation of BEISA signals with serum CA19-9 in the validation cohort to distinguish between HD and GC.

**Figure S24.** BEISA detects the expression level of a single circRNA marker. Overall and between-group *p* values were calculated similarly to those in the training cohort.

2. **Supplementary Tables.**

**Table S1.** DNA sequences of used in this assay. All the sequences of the strands used in the study are provided below (in 5’ to 3’ direction).

| **Name** | **Sequence** |
| --- | --- |
| **S1** | CACTGGTCAGCTCAGCAAATGTGATGTAGGTGGTAGAGGAACCGGAACCTACCGATAGCCGCTAGTTGCGGATGAAAAGATGGAGTGTGGTGTGAAGAACTGTGCCGGTTTGCTGA |
| **S2** | CCACACTTGCGACAGTCAATGTGATGTAGGTGGTAGAGGAATGCTGAGCTGACCAGTGTCAGCAAACCGGCACAGAAAGATGGAGTGTGGTGTGAAGAAGTGAAGAGTGTGCGTGC |
| **S3** | TCGCTGAGTAACCTGCCAATGTGATGTAGGTGGTAGAGGAAGACTGTCGCAAGTGTGGGCACGCACACTCTTCACAAAGATGGAGTGTGGTGTGAAGAAGCTCACGCACAAATCTG |
| **S4** | CTATCGGTAGGTTCCGGAATGTGATGTAGGTGGTAGAGGAAGGCAGGTTACTCAGCGACAGATTTGTGCGTGAGCAAAGATGGAGTGTGGTGTGAAGAATCATCCGCAACTAGCGG |
| **H1-URI1** | CTCTCTTGGCAGTTAG/i6FAMdT/GACCACGTTCTTTCCAGTTGCAGTAGGAAAGAACGTGG/iBHQ1dT/CACTAACTTTTCCTCTACCACCTACATCACA |
| **H2-URI1** | GGAAAGAACGTGGTCACTAACTGCCAAGAGAGAGTTAGTGACCACGTTCTTTCCTACTGCAACTTTTCTTCACACCACACTCCATCT |
| **H1-CWF19L1** | AGGTTTGCGAGGCTGCTTTGGA/i6FAMdT/GAGGAGAAGATCGTACTGACTTCTCCTCA/iBHQ1dT/CCAAAGCAGCCATTCCTCTACCACCTACATCACA |
| **H2-CWF19L1** | CTTCTCCTCATCCAAAGCAGCCTCGCAAACCTGGCTGCTTTGGATGAGGAGAAGTCAGTACGATTTTCTTCACACCACACTCCATCT |
| **H1-OGA** | GTGCTGAAGAATGAC/i6FAMdT/ACGACACCCTAACCACTGATCGAGTGGTGGTTAGGGTGTCG/iBHQ1dT/AGTCATAAACCTCTACCACCTACATCACA |
| **H2-OGA** | GTGGTTAGGGTGTCGTAGTCATTCTTCAGCACATGACTACGACACCCTAACCACCACTCGATCAAAACTTCACACCACACTCCATCT |
| **H1-YME1L1** | TCCAAACTCTCAACATCTGA/i6FAMdT/CCTCTGTCCCGAGCTGTGTCACGGGACAGAGGA/iBHQ1dT/CAGATGTTGTTTCCTCTACCACCTACATCACA |
| **H2-YME1L1** | CGGGACAGAGGATCAGATGTTGAGAGTTTGGACAACATCTGATCCTCTGTCCCGTGACACAGCTTTTCTTCACACCACACTCCATCT |
| **circ-URI1-SBM** | GUGGUCACUAAUUGCCAAGAGAGAAUCCAGGUAGGUUUAGUUGAGCACCGGAAAGAAC |
| **circ-URI1-DBM** | GUGGUCACUAAUUGCUAAGAGAGAAUCCAGGUAGGUUUAGUUGAGCACCGGAAAGAAC |
| **circ-URI1-TBM** | GUGGUCACUAAUUGCUAUGAGAGAAUCCAGGUAGGUUUAGUUGAGCACCGGAAAGAAC |
| **Capture probe-URI1** | SH(CH2)6-TTTTTTAAACCTACAGCCTGCTT |
| **Capture probe-CWF19L1** | SH(CH2)6-TTTTTAACCAGCAGGGTCCTGGA |
| **Capture probe-OGA** | SH(CH2)6-TTTTTGTATATTTTTGGTTTTCA |
| **Capture probe-YME1L1** | SH(CH2)6-TTTTTGGGTTCTTGGTCTCCAAT |
| **PrimerURI1-1** | GTCCTGCAGTCAATGCCTCT |
| **PrimerURI1-2** | GTCAGCCTTGTCCCTCCTTC |
| **PrimerCWF19L1-1** | ATGGCACAGAAACCGCTGC |
| **PrimerCWF19L1-2** | AGGTACACAATCTGCAGCCC |
| **PrimerOGA-1** | TGCAGAAGGAGAGTCAAGCG |
| **PrimerOGA-2** | TTGCTCAGCTTCCTCCACTG |
| **PrimerYME1L1-1** | TGCAACCCCAGGTTACAGTT |
| **PrimerYME1L1-2** | GGAGAAGGGTTCTGGACGGA |
| **Guide RNA-URI1** | UAGUGACCACGUUCUUUCCG |
| **linear-URI1** | GUGGUCACUAACUGCCAAGAGAGAAUCCAGCAUUGGUGAGUGAAAGAUGGUUUUAUUACACUAAGCAGGCUGUAGGUUUAGUUGAGCACCGGAAAGAAC |
| **circ-OGA** | GUGGUUAGGGUGUCGUAGUCAUUCUUCAGCACAAUUCUUAAUUGGAGACCAAGAACCC |
| **circ-YME1L1** | CGGGACAGAGGAUCAGAUGUUGAGAGUUUGGACAAACUCAUGAAAACCAAAAAUAUAC |
| **circ-CWF19L1** | CUUCUCCUCAUCCAAAGCAGCCUCGCAAACCUCCUCAGCCUCCAGGACCCUGCUGGUU |

**Table S2.** Recovery of four different concentrations of EVs using BESIA-based platforms.

| **Sample** | **Mode** | **Original (particles/ml)** | | **Measured (particles/ml)** | **Recovery(%)** | **RSD (%)** |
| --- | --- | --- | --- | --- | --- | --- |
| **1** | FL | | 10^7^ | 1.0×10^7^ | 102.7 | 8.8 |
|  | EC | | 10^7^ | 9.7×10^6^ | 97.0 | 9.5 |
|  |  | |  |  |  |  |
| **2** | FL | | 5×10^5^ | 4.8×10^5^ | 96.2 | 5.6 |
|  | EC | | 5×10^5^ | 5.2×10^5^ | 104.2 | 2.6 |
|  |  | |  |  |  |  |
| **3** | FL | | 5×10^6^ | 4.9×10^6^ | 97.7 | 6.5 |
|  | EC | | 5×10^6^ | 4.8×10^6^ | 95.2 | 3.6 |
|  |  | |  |  |  |  |
| **4** | FL | | 5×10^7^ | 5.3×10^7^ | 105.7 | 5.4 |
|  | EC | | 5×10^7^ | 5.5×10^7^ | 109.2 | 8.0 |

Note.“RSD”represents the relative standard deviation, RSD = (SD / Mean) × 100%.

**Table S3**: Clinical information on healthy donor (HD) and gastric cancer (GC) patients from the discovery cohort.

| **Index** | **Age** | **Stage** |
| --- | --- | --- |
| **Patient 1** | 80 | I |
| **Patient 2** | 59 | II |
| **Patient 3** | 57 | I |
| **Patient 4** | 60 | I |
| **Patient 5** | 58 | II |
| **Patient 6** | 47 | IV |
| **Patient 7** | 49 | III |
| **Patient 8** | 76 | IV |
| **Patient 9** | 73 | II |
| **Patient 10** | 52 | I |
| **Patient 11** | 36 | I |
| **Patient 12** | 70 | III |
| **Patient 13** | 51 | IV |
| **Patient 14** | 51 | I |
| **Patient 15** | 69 | III |
| **Patient 16** | 62 | IV |
| **Patient 17** | 75 | I |
| **Patient 18** | 46 | I |
| **Patient 19** | 54 | II |
| **Patient 20** | 51 | II |
| **HD1** | 48 | - |
| **HD2** | 26 | - |
| **HD3** | 44 | - |
| **HD4** | 57 | - |
| **HD5** | 61 | - |
| **HD6** | 54 | - |
| **HD7** | 50 | - |
| **HD8** | 48 | - |
| **HD9** | 65 | - |
| **HD10** | 58 | - |
| **HD11** | 56 | - |
| **HD12** | 36 | - |
| **HD13** | 43 | - |
| **HD14** | 53 | - |
| **HD15** | 49 | - |

**Table S4**: Clinical information on healthy donor (HD) and gastric cancer (GC) patients from the training cohort and validation cohort.

| **Index** | **Age** | **Stage** | **CEA** | **CA-199** |
| --- | --- | --- | --- | --- |
| **Patient 1** | 64 | IV | 8.86 | 493.64 |
| **Patient 2** | 73 | IV | 1.86 | 44.87 |
| **Patient 3** | 58 | III | 4.17 | 39.27 |
| **Patient 4** | 65 | III | 3.47 | 2.46 |
| **Patient 5** | 59 | III | 0.85 | 2.77 |
| **Patient 6** | 48 | II | 1.52 | 6.6 |
| **Patient 7** | 50 | II | 1.8 | 43.2 |
| **Patient 8** | 67 | I | 4.6 | 12.6 |
| **Patient 9** | 49 | II | 1.3 | 2.46 |
| **Patient 10** | 55 | II | 3.78 | 11.39 |
| **Patient 11** | 39 | I | 2.44 | 2.46 |
| **Patient 12** | 73 | I | 2.99 | 15.92 |
| **Patient 13** | 53 | I | 1.49 | 2.46 |
| **Patient 14** | 65 | III | 2.15 | 8.69 |
| **Patient 15** | 59 | IV | 4.11 | 4.94 |
| **Patient 16** | 53 | III | 2.45 | 5.39 |
| **Patient 17** | 48 | IV | 4.19 | 28.53 |
| **Patient 18** | 39 | I | 1.15 | 9.35 |
| **Patient 19** | 53 | III | 3.49 | 14.36 |
| **Patient 20** | 26 | II | 1.6 | 20.94 |
| **Patient 21** | 58 | III | 3.38 | 3.89 |
| **Patient 22** | 70 | III | 3.6 | 22.69 |
| **Patient 23** | 56 | II | 1.27 | 8.59 |
| **Patient 24** | 49 | I | 5.28 | 2.46 |
| **Patient 25** | 59 | III | 9.53 | 2.46 |
| **Patient 26** | 69 | III | 8.76 | 35.85 |
| **Patient 27** | 76 | IV | 1.83 | 40.22 |
| **Patient 28** | 37 | I | 1.83 | 59.87 |
| **Patient 29** | 51 | I | 1.13 | 3.58 |
| **Patient 30** | 68 | I | 1.12 | 3.14 |
| **Patient 31** | 55 | II | 2.56 | 1.29 |
| **Patient 32** | 57 | I | 67 | 4.9 |
| **Patient 33** | 65 | I | 2.31 | 2.53 |
| **Patient 34** | 60 | I | 2.55 | 6.58 |
| **Patient 35** | 81 | I | 1.67 | 7.82 |
| **Patient 36** | 51 | I | 0.79 | 1.21 |
| **Patient 37** | 69 | I | 1.49 | 8.74 |
| **Patient 38** | 60 | II | 1.36 | 7.14 |
| **Patient 39** | 52 | II | 1.41 | 77.95 |
| **Patient 40** | 61 | II | 1.12 | 8.99 |
| **Patient 41** | 45 | III | 1.9 | 9.62 |
| **Patient 42** | 42 | IV | 37.05 | 32.4 |
| **Patient 43** | 57 | IV | 232.46 | 99.03 |
| **Patient 44** | 67 | IV | 38.67 | 0.23 |
| **Patient 45** | 75 | IV | 447.83 | 2.98 |
| **Patient 46** | 57 | IV | 1.26 | 1.26 |
| **Patient 47** | 76 | IV | 6.04 | 0.98 |
| **Patient 48** | 55 | IV | 1.58 | 0.98 |
| **Patient 49** | 79 | III | 1.54 | 8.59 |
| **Patient 50** | 63 | III | 63 | 3.69 |
| **HD1** | 41 | _ | 3.52 | 3.22 |
| **HD2** | 40 | _ | 1.31 | 11.6 |
| **HD3** | 36 | _ | 0.74 | 4.65 |
| **HD4** | 39 | _ | 2.2 | 2.46 |
| **HD5** | 37 | _ | 1.14 | 5.16 |
| **HD6** | 34 | _ | 1.76 | 2.46 |
| **HD7** | 49 | _ | 2.95 | 32.84 |
| **HD8** | 37 | _ | 0.85 | 2.46 |
| **HD9** | 33 | _ | 1.42 | 24.18 |
| **HD10** | 31 | _ | 2.06 | 16.18 |
| **HD11** | 30 | _ | 0.48 | 22.8 |
| **HD12** | 24 | _ | 1.52 | 17.1 |
| **HD13** | 36 | _ | 0.99 | 9.37 |
| **HD14** | 29 | _ | 0.97 | 3.7 |
| **HD15** | 38 | _ | 1.33 | 34.9 |
| **HD16** | 54 | _ | 2.35 | 15.68 |
| **HD17** | 54 | _ | 0.54 | 9.49 |
| **HD18** | 38 | _ | 0.78 | 7.22 |
| **HD19** | 26 | _ | 2.13 | 15.77 |
| **HD20** | 44 | _ | 1.55 | 2.46 |
| **HD21** | 41 | _ | 0.96 | 2.46 |
| **HD22** | 31 | _ | 19.27 | 19.27 |
| **HD23** | 41 | _ | 1.11 | 8.63 |
| **HD24** | 44 | _ | 2.39 | 2.46 |
| **HD25** | 48 | _ | 0.91 | 2.46 |
